# *N*-[4-Acetyl-5-(4-fluoro­phen­yl)-4,5-di­hydro-1,3,4-thia­diazol-2-yl]acetamide

**DOI:** 10.1107/S1600536813009367

**Published:** 2013-04-13

**Authors:** H. D. Kavitha, Sheetal B. Marganakop, Ravindra R. Kamble, K. R. Roopashree, H. C. Devarajegowda

**Affiliations:** a Department of Physics, Government Science College, Hassan 573 201, Karnataka, India; b Department of Studies in Chemistry, Karnataka University, Dharwad 580 003, Karnataka , India; c Department of Physics, Yuvaraja’s College (Constituent College), University of Mysore, Mysore 570 005, Karnataka, India

## Abstract

The title mol­ecule, C_12_H_12_FN_3_O_2_S, shows a short intra­molecular S⋯O contact of 2.682 (18) Å. The dihedral angle between the thia­diazole ring and the benzene ring is 86.82 (11)°. In the crystal, N—H⋯O and C—H⋯O hydrogen bonds generate an *R*_2_^1^(6) graph-set motif between adjacent mol­ecules. Pairs of futher C—H⋯O hydrogen bonds form inversion dimers with *R*_2_^2^(8) ring motifs. These combine to generate a three-dimensional network and stack the mol­ecules along the *b* axis.

## Related literature

For biological applications of 1,3,4-thia­diazole derivatives, see: Matysiak & Opolski (2006 ▶); Kumar *et al.* (2012 ▶); Oruç *et al.* (2004 ▶); Kadi *et al.* (2007 ▶); Noolvi *et al.* (2011 ▶); Matysiak *et al.* (2006 ▶); Marganakop *et al.* (2012 ▶). For a related structure, see: Zhang (2009 ▶). For graph-set notation, see: Bernstein *et al.* (1995 ▶).
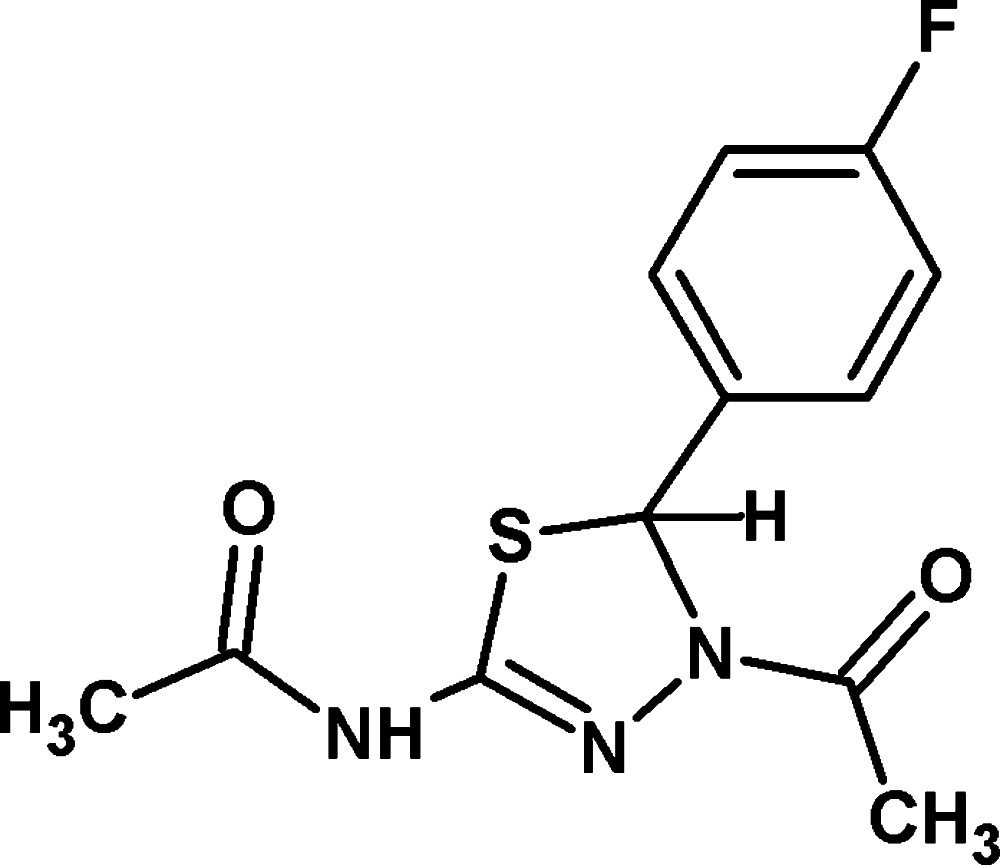


## Experimental

### 

#### Crystal data

C_12_H_12_FN_3_O_2_S
*M* *_r_* = 281.31
Monoclinic, 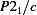

*a* = 9.5061 (6) Å
*b* = 11.2152 (7) Å
*c* = 12.7752 (7) Å
β = 101.823 (4)°
*V* = 1333.11 (14) Å^3^
*Z* = 4
Mo *K*α radiation
μ = 0.26 mm^−1^
*T* = 296 K
0.24 × 0.20 × 0.12 mm

#### Data collection

Bruker SMART CCD area-detector diffractometer
Absorption correction: multi-scan (*SADABS*; Sheldrick, 2007 ▶) *T*_min_ = 0.770, *T*_max_ = 1.000
11372 measured reflections
2352 independent reflections
2035 reflections with *I* > 2σ(*I*)
*R*_int_ = 0.024

#### Refinement

*R*[*F* ^2^ > 2σ(*F* ^2^)] = 0.041
*wR*(*F* ^2^) = 0.121
*S* = 1.07
2352 reflections
172 parameters
H-atom parameters constrained
Δρ_max_ = 0.42 e Å^−3^
Δρ_min_ = −0.34 e Å^−3^

### 

Data collection: *SMART* (Bruker, 2001 ▶); cell refinement: *SAINT* (Bruker, 2001 ▶); data reduction: *SAINT*; program(s) used to solve structure: *SHELXS97* (Sheldrick, 2008 ▶); program(s) used to refine structure: *SHELXL97* (Sheldrick, 2008 ▶); molecular graphics: *ORTEP-3 for Windows* (Farrugia, 2012 ▶); software used to prepare material for publication: *SHELXL97*.

## Supplementary Material

Crystal structure: contains datablock(s) I, global. DOI: 10.1107/S1600536813009367/sj5314sup1.cif

Structure factors: contains datablock(s) I. DOI: 10.1107/S1600536813009367/sj5314Isup2.hkl

Supplementary material file. DOI: 10.1107/S1600536813009367/sj5314Isup3.cml

Additional supplementary materials:  crystallographic information; 3D view; checkCIF report

## Figures and Tables

**Table 1:** Hydrogen-bond geometry (Å, °)

*D*—H⋯*A*	*D*—H	H⋯*A*	*D*⋯*A*	*D*—H⋯*A*
N5—H5⋯O4^i^	0.86	1.96	2.815 (2)	171
C10—H10⋯O3^ii^	0.93	2.58	3.267 (3)	131
C17—H17*A*⋯O4^i^	0.96	2.46	3.316 (3)	148
C19—H19*B*⋯O4^iii^	0.96	2.55	3.335 (3)	139

Symmetry codes: (i) 
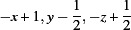
; (ii) 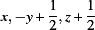; (iii) 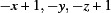.

**Fig. 1.**
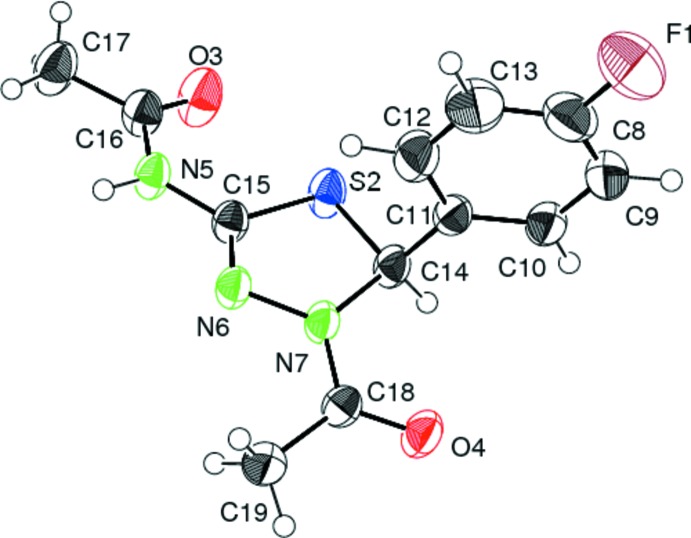
The molecular structure of the title compound. Displacement ellipsoids are drawn at the 50% probability level. Hydrogen atoms are shown as spheres of arbitrary radius.

**Fig. 2.**
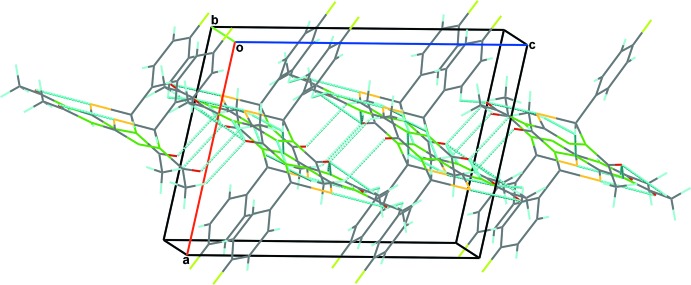
Packing of the molecule in the unit cell.

## supplementary crystallographic information

### Comment

1,3,4-Thiadiazole derivatives are of great importance to chemists as well as biologists as they are found in a large variety of naturally occurring compounds and also pharmacologically potent molecules. These derivatives are known to exhibit a broad spectrum of activities including antiproliferative, antituberculosis, anti-inflammatory, anticancer and antimicrobial activities (Matysiak *et al.*, 2006; Kumar *et al.*, 2012; Oruç *et al.*, 2004; Kadi *et al.*, 2007; Noolvi *et al.*, 2011; Matysiak & Opolski, 2006; Marganakop *et al.*, 2012).

The asymmetric unit of the structure of *N*-[4-Acetyl-5-(4-fluorophenyl)-4,5-dihydro-1,3,4-thiadiazol-2-yl] -acetamide is shown in Fig. 1 and exhibits a short intramolecular S2···O3 contact of 2.682 (18) Å. The dihedral angle between the thiadiazole ring (S2/N6/N7/C14/C15) and the benzene ring (C8–C13) is 86.82 (11)°. In the structure, all bond lengths and angles are within normal ranges (Zhang, 2009).

In the crystal, the N5—H5···O4 and C17—H17A···O4 hydrogen bonds (Table 1) link adjacent molecules forming rings with an *R*^1^_2_(6) graph-set motif (Bernstein *et al.*, 1995). The crystal structure is further stabilized by other intermolecular C—H···O hydrogen bonds, (Table 1), that generate inversion dimers with *R*^2^_2_(8) ring motifs. The overall crystal packing components generate a three-dimensional network, stacking molecules along the *b* axis, (Fig. 2).

### Experimental

A mixture of *p*-fluorobenzaldehyde (0.005 mole), and thiosemicarbazide (0.005 mole) was refluxed in ethanol (10 ml) and acetic acid (2 drops), after completion of the reaction the resulting pale yellow powder was filtered, dried and crystallized in ethanol to obtain (*E*)-1-(4-fluoro benzylidene)thiosemicarbazide, which was further heated at 80–90°C for about 4 hrs and the reaction mixture was cooled to room temperature and poured into ice cold water. The precipitate obtained was filtered off, washed with water, dried and purified by crystallization in aqueous alcohol (80%, *v*/*v*) to yield pale yellow crystals of N– [4-acetyl-5-(4-fluorophenyl)-4,5-dihydro-[1,3,4]thiadiazol-2-yl]- acetamide. Yield: (70%), m. p: 490 K.

### Refinement

All H atoms were positioned at calculated positions, N—H = 0.86 Å, C—H = 0.93 Å for aromatic H, C—H = 0.98 Å for methine H and C—H = 0.96 Å for methyl H and refined using a riding model with *U*_iso_(H) = 1.5*U*_eq_(C) for methyl H and *U*_iso_(H) = 1.2*U*_eq_(C, N) for aromatic, methine and amide H.

### Crystal data

**Table:**

C_12_H_12_FN_3_O_2_S	*F*(000) = 584
*M**_r_* = 281.31	*D*_x_ = 1.402 Mg m^−^^3^
Monoclinic, *P*2_1_/*c*	Melting point: 490 K
Hall symbol: -P 2ybc	Mo *K*α radiation, λ = 0.71073 Å
*a* = 9.5061 (6) Å	Cell parameters from 2352 reflections
*b* = 11.2152 (7) Å	θ = 2.2–25.0°
*c* = 12.7752 (7) Å	µ = 0.26 mm^−^^1^
β = 101.823 (4)°	*T* = 296 K
*V* = 1333.11 (14) Å^3^	Plate, colourless
*Z* = 4	0.24 × 0.20 × 0.12 mm

### Data collection

**Table:**

Bruker SMART CCD area-detector diffractometer	2352 independent reflections
Radiation source: fine-focus sealed tube	2035 reflections with *I* > 2σ(*I*)
Graphite monochromator	*R*_int_ = 0.024
ω and φ scans	θ_max_ = 25.0°, θ_min_ = 2.2°
Absorption correction: multi-scan (*SADABS*; Sheldrick, 2007)	*h* = −11→11
*T*_min_ = 0.770, *T*_max_ = 1.000	*k* = −13→12
11372 measured reflections	*l* = −15→15

### Refinement

**Table:**

Refinement on *F*^2^	Primary atom site location: structure-invariant direct methods
Least-squares matrix: full	Secondary atom site location: difference Fourier map
*R*[*F*^2^ > 2σ(*F*^2^)] = 0.041	Hydrogen site location: inferred from neighbouring sites
*wR*(*F*^2^) = 0.121	H-atom parameters constrained
*S* = 1.07	*w* = 1/[σ^2^(*F*_o_^2^) + (0.0619*P*)^2^ + 0.7142*P*] where *P* = (*F*_o_^2^ + 2*F*_c_^2^)/3
2352 reflections	(Δ/σ)_max_ < 0.001
172 parameters	Δρ_max_ = 0.42 e Å^−^^3^
0 restraints	Δρ_min_ = −0.34 e Å^−^^3^

### Special details

**Table:**

Experimental. Spectroscopic data IR (KBr); 3233, 2799, 1646, 1626, 1H NMR (300 MHz, CDCl3, δ p.p.m.): 2.11 (s, 3H, CH3 of NHCOCH3), 2.24 (s, 3H, CH3 of –NCOCH3), 4.70 (s, 1H, C—H of C5—H), 6.85–7.10 (m, 4H, Ar—H), 11.77 (s, 1H, NHCO), MS (m/*z*, 70 eV); 282 (*M*^+1^, 20), 239 (26), 204 (100).
Geometry. All s.u.'s (except the s.u. in the dihedral angle between two l.s. planes) are estimated using the full covariance matrix. The cell s.u.'s are taken into account individually in the estimation of s.u.'s in distances, angles and torsion angles; correlations between s.u.'s in cell parameters are only used when they are defined by crystal symmetry. An approximate (isotropic) treatment of cell s.u.'s is used for estimating s.u.'s involving l.s. planes.
Refinement. Refinement of *F*^2^ against ALL reflections. The weighted *R*-factor *wR* and goodness of fit *S* are based on *F*^2^, conventional *R*-factors *R* are based on *F*, with *F* set to zero for negative *F*^2^. The threshold expression of *F*^2^ > 2σ(*F*^2^) is used only for calculating *R*-factors(gt) *etc*. and is not relevant to the choice of reflections for refinement. *R*-factors based on *F*^2^ are statistically about twice as large as those based on *F*, and *R*- factors based on ALL data will be even larger.

### Fractional atomic coordinates and isotropic or equivalent isotropic displacement parameters (Å^2^)

**Table:**

	*x*	*y*	*z*	*U*_iso_*/*U*_eq_	
F1	−0.11204 (19)	0.1673 (2)	0.41894 (18)	0.1026 (7)	
S2	0.32591 (7)	0.09255 (5)	0.09191 (4)	0.0449 (2)	
O3	0.2686 (2)	−0.01822 (15)	−0.09810 (14)	0.0665 (5)	
O4	0.56411 (17)	0.11842 (14)	0.42490 (13)	0.0472 (4)	
N5	0.3591 (2)	−0.14036 (15)	0.03899 (13)	0.0407 (4)	
H5	0.3826	−0.2127	0.0571	0.049*	
N6	0.45429 (18)	−0.08120 (15)	0.21164 (13)	0.0367 (4)	
N7	0.46451 (18)	0.01994 (15)	0.27693 (14)	0.0374 (4)	
C8	0.0057 (3)	0.1564 (3)	0.3747 (2)	0.0626 (7)	
C9	0.0942 (3)	0.2522 (2)	0.3768 (2)	0.0558 (6)	
H9	0.0750	0.3239	0.4076	0.067*	
C10	0.2141 (2)	0.2394 (2)	0.33124 (17)	0.0441 (5)	
H10	0.2768	0.3032	0.3321	0.053*	
C11	0.2413 (2)	0.13353 (19)	0.28503 (16)	0.0372 (5)	
C12	0.1473 (3)	0.0389 (2)	0.2839 (2)	0.0551 (6)	
H12	0.1645	−0.0327	0.2520	0.066*	
C13	0.0287 (3)	0.0497 (3)	0.3296 (3)	0.0677 (8)	
H13	−0.0340	−0.0140	0.3297	0.081*	
C14	0.3719 (2)	0.12171 (18)	0.23621 (16)	0.0375 (5)	
H14	0.4283	0.1953	0.2490	0.045*	
C15	0.3864 (2)	−0.05435 (18)	0.11717 (16)	0.0358 (5)	
C16	0.2973 (3)	−0.1186 (2)	−0.06565 (17)	0.0453 (5)	
C17	0.2681 (3)	−0.2264 (2)	−0.1344 (2)	0.0604 (7)	
H17A	0.2994	−0.2961	−0.0925	0.091*	
H17B	0.3192	−0.2206	−0.1917	0.091*	
H17C	0.1669	−0.2321	−0.1635	0.091*	
C18	0.5596 (2)	0.02706 (18)	0.37069 (16)	0.0363 (5)	
C19	0.6546 (3)	−0.0775 (2)	0.40480 (18)	0.0478 (6)	
H19A	0.6340	−0.1389	0.3515	0.072*	
H19B	0.6377	−0.1074	0.4716	0.072*	
H19C	0.7533	−0.0536	0.4134	0.072*	

### Atomic displacement parameters (Å^2^)

**Table:**

	*U*^11^	*U*^22^	*U*^33^	*U*^12^	*U*^13^	*U*^23^
F1	0.0620 (10)	0.1312 (19)	0.1269 (17)	0.0023 (11)	0.0478 (11)	−0.0189 (15)
S2	0.0679 (4)	0.0285 (3)	0.0365 (3)	0.0074 (2)	0.0066 (3)	0.0035 (2)
O3	0.1095 (15)	0.0395 (10)	0.0444 (9)	0.0104 (10)	0.0014 (9)	0.0058 (8)
O4	0.0573 (9)	0.0333 (8)	0.0485 (9)	−0.0053 (7)	0.0049 (7)	−0.0088 (7)
N5	0.0608 (11)	0.0261 (9)	0.0339 (9)	0.0035 (8)	0.0064 (8)	0.0009 (7)
N6	0.0464 (10)	0.0272 (9)	0.0352 (9)	0.0023 (7)	0.0055 (7)	−0.0024 (7)
N7	0.0465 (10)	0.0263 (9)	0.0373 (9)	0.0032 (7)	0.0038 (7)	−0.0032 (7)
C8	0.0439 (13)	0.082 (2)	0.0635 (16)	0.0081 (13)	0.0142 (12)	−0.0031 (15)
C9	0.0545 (14)	0.0586 (16)	0.0519 (14)	0.0122 (12)	0.0057 (11)	−0.0124 (12)
C10	0.0479 (12)	0.0374 (12)	0.0440 (12)	0.0021 (9)	0.0022 (9)	−0.0049 (10)
C11	0.0425 (11)	0.0306 (11)	0.0355 (10)	0.0022 (9)	0.0007 (8)	0.0009 (8)
C12	0.0569 (14)	0.0389 (13)	0.0706 (17)	−0.0043 (11)	0.0157 (12)	−0.0055 (12)
C13	0.0537 (15)	0.0634 (18)	0.089 (2)	−0.0146 (13)	0.0225 (14)	−0.0058 (16)
C14	0.0481 (11)	0.0247 (10)	0.0381 (11)	0.0007 (9)	0.0051 (9)	0.0005 (8)
C15	0.0451 (11)	0.0272 (10)	0.0352 (11)	0.0004 (9)	0.0083 (9)	0.0008 (8)
C16	0.0603 (14)	0.0361 (12)	0.0377 (11)	0.0047 (10)	0.0060 (10)	0.0030 (10)
C17	0.0867 (18)	0.0469 (15)	0.0405 (13)	0.0070 (13)	−0.0036 (12)	−0.0061 (11)
C18	0.0389 (10)	0.0315 (11)	0.0387 (11)	−0.0051 (8)	0.0082 (8)	−0.0007 (9)
C19	0.0494 (13)	0.0440 (14)	0.0452 (13)	0.0053 (10)	−0.0014 (10)	−0.0028 (10)

### Geometric parameters (Å, º)

**Table:**

F1—C8	1.359 (3)	C10—C11	1.374 (3)
S2—C15	1.753 (2)	C10—H10	0.9300
S2—C14	1.835 (2)	C11—C12	1.386 (3)
O3—C16	1.211 (3)	C11—C14	1.505 (3)
O4—C18	1.233 (3)	C12—C13	1.376 (4)
N5—C16	1.368 (3)	C12—H12	0.9300
N5—C15	1.374 (3)	C13—H13	0.9300
N5—H5	0.8600	C14—H14	0.9800
N6—C15	1.283 (3)	C16—C17	1.487 (3)
N6—N7	1.399 (2)	C17—H17A	0.9600
N7—C18	1.347 (3)	C17—H17B	0.9600
N7—C14	1.470 (3)	C17—H17C	0.9600
C8—C9	1.361 (4)	C18—C19	1.490 (3)
C8—C13	1.365 (4)	C19—H19A	0.9600
C9—C10	1.389 (3)	C19—H19B	0.9600
C9—H9	0.9300	C19—H19C	0.9600
			
C15—S2—C14	88.91 (9)	N7—C14—S2	102.66 (13)
C16—N5—C15	124.35 (18)	C11—C14—S2	112.67 (14)
C16—N5—H5	117.8	N7—C14—H14	109.1
C15—N5—H5	117.8	C11—C14—H14	109.1
C15—N6—N7	109.31 (17)	S2—C14—H14	109.1
C18—N7—N6	122.01 (17)	N6—C15—N5	120.17 (19)
C18—N7—C14	120.85 (17)	N6—C15—S2	118.48 (16)
N6—N7—C14	117.06 (16)	N5—C15—S2	121.34 (15)
F1—C8—C9	118.6 (3)	O3—C16—N5	121.5 (2)
F1—C8—C13	118.1 (3)	O3—C16—C17	123.5 (2)
C9—C8—C13	123.3 (2)	N5—C16—C17	115.0 (2)
C8—C9—C10	117.8 (2)	C16—C17—H17A	109.5
C8—C9—H9	121.1	C16—C17—H17B	109.5
C10—C9—H9	121.1	H17A—C17—H17B	109.5
C11—C10—C9	120.8 (2)	C16—C17—H17C	109.5
C11—C10—H10	119.6	H17A—C17—H17C	109.5
C9—C10—H10	119.6	H17B—C17—H17C	109.5
C10—C11—C12	119.2 (2)	O4—C18—N7	119.26 (19)
C10—C11—C14	119.87 (19)	O4—C18—C19	122.74 (19)
C12—C11—C14	120.9 (2)	N7—C18—C19	118.00 (18)
C13—C12—C11	120.7 (2)	C18—C19—H19A	109.5
C13—C12—H12	119.6	C18—C19—H19B	109.5
C11—C12—H12	119.6	H19A—C19—H19B	109.5
C8—C13—C12	118.1 (3)	C18—C19—H19C	109.5
C8—C13—H13	120.9	H19A—C19—H19C	109.5
C12—C13—H13	120.9	H19B—C19—H19C	109.5
N7—C14—C11	114.02 (17)		
			
C15—N6—N7—C18	−162.76 (18)	C12—C11—C14—N7	−53.9 (3)
C15—N6—N7—C14	14.0 (2)	C10—C11—C14—S2	−117.30 (19)
F1—C8—C9—C10	179.9 (2)	C12—C11—C14—S2	62.6 (2)
C13—C8—C9—C10	−0.5 (4)	C15—S2—C14—N7	15.35 (14)
C8—C9—C10—C11	0.5 (4)	C15—S2—C14—C11	−107.73 (16)
C9—C10—C11—C12	0.1 (3)	N7—N6—C15—N5	−178.19 (17)
C9—C10—C11—C14	180.0 (2)	N7—N6—C15—S2	0.2 (2)
C10—C11—C12—C13	−0.8 (4)	C16—N5—C15—N6	−174.9 (2)
C14—C11—C12—C13	179.4 (2)	C16—N5—C15—S2	6.8 (3)
F1—C8—C13—C12	179.5 (3)	C14—S2—C15—N6	−10.22 (18)
C9—C8—C13—C12	−0.1 (5)	C14—S2—C15—N5	168.13 (18)
C11—C12—C13—C8	0.7 (4)	C15—N5—C16—O3	4.0 (4)
C18—N7—C14—C11	−81.0 (2)	C15—N5—C16—C17	−175.6 (2)
N6—N7—C14—C11	102.1 (2)	N6—N7—C18—O4	179.87 (18)
C18—N7—C14—S2	156.79 (16)	C14—N7—C18—O4	3.2 (3)
N6—N7—C14—S2	−20.1 (2)	N6—N7—C18—C19	−0.6 (3)
C10—C11—C14—N7	126.2 (2)	C14—N7—C18—C19	−177.34 (18)

### Hydrogen-bond geometry (Å, º)

**Table:**

*D*—H···*A*	*D*—H	H···*A*	*D*···*A*	*D*—H···*A*
N5—H5···O4^i^	0.86	1.96	2.815 (2)	171
C10—H10···O3^ii^	0.93	2.58	3.267 (3)	131
C17—H17*A*···O4^i^	0.96	2.46	3.316 (3)	148
C19—H19*B*···O4^iii^	0.96	2.55	3.335 (3)	139

Symmetry codes: (i) −*x*+1, *y*−1/2, −*z*+1/2; (ii) *x*, −*y*+1/2, *z*+1/2; (iii) −*x*+1, −*y*, −*z*+1.
